# Assessing Continuity of Adherence to Precautionary Measures for COVID-19 among Vaccinated People in Jazan, Saudi Arabia

**DOI:** 10.3390/microorganisms11030800

**Published:** 2023-03-21

**Authors:** Anwar Alameer, Yahya Maslamani, Ibrahim M. Gosadi, Mohammed Y. Elamin, Mohammed A. Muaddi, Ahmad Y. Alqassim, Abrar Doweri, Ibrahim Namis, Fatimah Busayli, Hussam Ahmadini, Yehya Hejri, Abdu Dahlan

**Affiliations:** 1 Public Health Administration, Jazan Health Directorate, Jazan 82611, Saudi Arabia; 2 Department of Family and Community Medicine, Faculty of Medicine, Jazan University, Jazan 45142, Saudi Arabia; 3 Endocrinology and Diabetic Center, Jazan Health Directorate, Jazan 82611, Saudi Arabia; 4 Sabya General Hospital, Jazan Health Directorate, Jazan 82611, Saudi Arabia

**Keywords:** COVID-19, respiratory hygiene, variants, post-vaccination, adherence, prevention

## Abstract

Background: Adherence to behavioral respiratory hygiene practices is essential in preventing the transmission of COVID-19, especially given the appearance of new variants of the COVID-19 virus. This study estimated the pre- and post-vaccination levels of adherence to COVID-19 preventive behavioral measures among vaccinated people. Methods: This cross-sectional study assessed the sociodemographics and preventive behavioral measures, and pre- and post-vaccination data, via a questionnaire. Paired t-tests and Chi-squared tests were used to assess the variation in adherence levels. Results: Of the 480 participants, 57.9% were male, and 30.4% were aged between 30 and 39 years of age. After vaccination, there was a statistically significant decline in adherence to all the assessed behavioral protective measures (*p* < 0.05). Being 50 years old or older, female, a healthcare worker, and a smoker were associated with higher adherence levels compared with other groups in the same categories. Conclusions: A change in the behavior of the community members regarding COVID-19 after receiving the vaccination and a reduction in adherence to respiratory hygiene practices was observed. This indicates the importance of raising awareness about the possibility of reinfection with COVID-19 despite the vaccination, and the importance of behavioral respiratory hygiene for the prevention and control of COVID-19.

## 1. Introduction

Coronavirus disease 2019 (COVID-19) was first discovered in Wuhan, China, in December 2019. The World Health Organization (WHO) declared a pandemic in March 2020 after it had spread to more than 200 territories. As of 5 February 2023, 754 million confirmed cases and 6.8 million deaths have been reported globally [1]. About 829,388 confirmed coronavirus cases and 9614 deaths were recorded in Saudi Arabia by February 2023. The curve of the coronavirus epidemic, which fluctuates across years and seasons, is closely monitored by the Saudi Ministry of Health in terms of recorded cases and deaths for the sake of urgent intervention and control. To date, more than 68 million coronavirus vaccine doses have been administered in Saudi Arabia [2].

As of the start of 2023, despite the preventive and control measures applied against COVID-19, infections with the virus are ongoing. In the period between 12 and 19 January 2023, 1.3 million new cases of COVID-19 were reported in the Western Pacific region. Similarly, 649,000 and 234,000 new cases of COVID-19 were reported in the same week in the Americas and Europe, respectively [3]. These infection rates are augmented by the likely appearance of new variants of the virus due to the constant changes in its properties. The WHO recommended the use of letters of the Greek alphabet to indicate new variants of concern of SARS-CoV-2, which causes COVID-19. Currently, there are five main variants of concern, namely, alpha, beta, gamma, delta, and omicron. Each variant’s first appearance was linked to a specific country, while the first detected cases of the omicron variant were documented in several countries [4].

Although vaccines are among the interventions that have saved millions of lives since their development, behavioral precautions are still considered a cornerstone in suppressing the exponential growth in COVID-19 cases [5]. Additionally, strict policies (e.g., behavioral precautions, travel restrictions, lockdowns, and curfews) for the prevention and control of COVID-19 have been applied to several populations. Many regulations have diminished as a response to an improvement in the epidemic curve of COVID-19 cases, with careful continuation of regular assessments of the epidemiological situation. For example, on 23 March 2020, the United Kingdom’s government announced the first lockdown in the UK, which was followed by conditional lifting of the lockdown in the subsequent months, until the final removal of most legal limits on social contact by July 2021 [6]. Nonetheless, the ongoing incidence of COVID-19 infections despite vaccinations indicates the importance of adhering to respiratory hygiene practices for the prevention and control of respiratory illnesses, including COVID-19.

Respiratory hygiene is a preventive behavioral precaution that limits the transmission of respiratory pathogens, including COVID-19, via airborne or droplet routes. Respiratory hygiene includes several elements, such as covering the nose and mouth when coughing or sneezing, using and appropriately disposing of tissues, practicing appropriate hand washing and sanitization, avoiding touching the nose and mouth, and wearing face masks in individuals exhibiting respiratory symptoms [7].

Despite the reported effectiveness of COVID-19 vaccinations in preventing infection, the risk of breakthrough infections is considerable, elevating the importance of adhering to behavioral precautions against COVID-19. Adherence to behavioral precautions is essential for preventing the transmission of COVID-19 because it helps the population return to normal activities [8,9]. Vaccinations and adherence to behavioral precautions synergize to achieve a professional control strategy.

There are multiple conceptual frameworks that can be correlated with the changes in personal protective behavior concerning COVID-19. Nonetheless, due to the nature of the disease and the associated factors influencing adherence to personal protective behavior, the conceptual framework of the current investigation was associated with the health belief model. The model assumes that optimal behavior toward a particular disease is affected by perceived susceptibility, severity, barriers, and benefits.

Studies assessing the adherence of individuals vaccinated against COVID-19 to respiratory behavioral precautions are limited in Saudi Arabia. The main objective of this study was to estimate the pre- and post-vaccination levels of adherence to COVID-19 preventive measures among vaccinated people in the Jazan area, and to explore the associated factors. This estimation is essential for assessing and analyzing the current situation and for determining beneficial control strategies for future prevention. The assessment of the factors associated with the adherence of the participants concerning personal protective behavior is followed by the correlation of the findings with the health belief model to allow a better explanation of the factors that might predict the continuity of adherence to precautionary measures, especially regarding diseases of a respiratory nature.

## 2. Materials and Methods

### 2.1. Study Design and Settings

**Figure 1:**
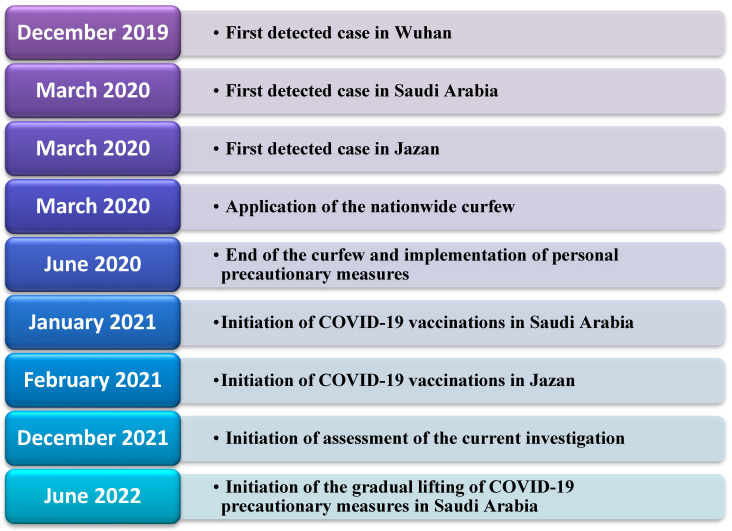
Timeline of COVID-19 prevention and control measures in the setting of Jazan, Saudi Arabia.

This cross-sectional study was conducted in the Jazan region of southwest Saudi Arabia. Jazan Health Affairs offers its services to more than almost 1.4 million people in 14 cities and numerous villages. Figure 1 illustrates the timeline of COVID-19 in the region and the performance of the assessment in the current investigation. The target population included all vaccinated people from February 2021 to July 2021. This investigation was performed after securing ethical approval from the Jazan Health Ethics Committee (approval number 2184, dated October 2021). Informed consent was secured from the study participants before enrolment, and the study was performed according to the principles of the Declaration of Helsinki.

### 2.2. Study Population and Data Collection Procedures

In total, there were approximately 715,579 vaccinated people in the 14 administrative areas at the start of the study. The participants were chosen according to the following eligibility criteria: aged ≥ 18 years, living in the Jazan region, and having taken the COVID-19 vaccine. Participants were excluded if they refused to give informed consent, were younger than 18 years, did not take the vaccine, and did not reside in the Jazan region.

Participants were chosen randomly from eight vaccination data banks of the public health sector, and the desired sample size was proportional to the size of each area’s vaccinated population; therefore, eligible participants were recruited by a simple randomization method proportional to the number of vaccinated people in each city. Participants were contacted by mobile phone and recruited voluntarily after receiving a complete description of the study. Then they were filtered by the study’s inclusion and exclusion criteria. On average, it took 20 min to complete the questionnaire. Data collection was completed within a period of 2 months from the study’s initiation. According to the following parameters, there was an estimated adherence level of 50% among the population of Jazan, a 0.05 margin of error, and a 95% confidence interval. The sample size was calculated using the method of Swanson and Cohen (N = Z2P (1 − P)/d2). The calculated sample size was 384 subjects, which was adjusted to 422 to account for a 10% non-response rate.

### 2.3. Measures

This study evaluated differences in pre- and post-vaccination levels of adherence to COVID-19 preventive measures among vaccinated people and explored the associated factors. The dependent variables were differences between pre- and post-vaccination levels of adherence to COVID-19 preventive measures among vaccinated people in the Jazan area. Data concerning adherence during the pre- and post-vaccination stages were collected at one time point. The independent variables were age, gender, area of residence, economic status, social status, education level, employment type (medical vs. non-medical), smoking status, and having a chronic illness.

The data collection tool of the International Citizen Project COVID-19 general questionnaire was used in this study. It comprised three parts that assessed the level of adherence to COVID-19 preventive measures and the associated factors according to the WHO guidelines and the Saudi national guidelines on COVID-19 prevention [10]. The first part of the questionnaire collected sociodemographic information about each participant. The second part assessed daily and professional life during the coronavirus epidemic, while the third part considered personal and community preventive measures. Adherence to preventive measures was evaluated using 10 “yes” or “no” questions. It included the following: the use of a face mask, physical distancing, coughing or sneezing into the crease of the elbow or covering the mouth and nose with a disposable handkerchief, hand washing/sanitizing immediately after coughing or sneezing, checking body temperature at least twice a week, regular hand washing during the day, using alcohol-based hand sanitizer during the day, avoiding touching the face (eyes, nose, mouth), sanitizing phones when returning home, and staying at home when experiencing influenza-like symptoms. The internal consistency of the items was tested via a Cronbach’s alpha test, revealing a value of 0.85. Additionally, the external consistency of the items was tested via the kappa test, revealing an overall value of 0.86.

### 2.4. Statistical Analysis

Data were analyzed using the Statistical Package for the Social Sciences, version 23. The analysis of the data was performed via three main steps. The first step included performing descriptive statistics. The descriptive statistics were used to summarize the main findings of the study, including the frequencies, proportions, means, and standard deviations. The descriptive analysis was followed up by conducting inferential statistics to identify the factors associated with adherence levels. Finally, factors identified to be associated with adherence levels were further assessed via the health belief model.

A scoring system was utilized for the assessment of adherence. Each adherence item scored 1 if the study participant confirmed that they adhered to the measure and 0 otherwise. Adherence scores were calculated by summing the responses to the 10 questions related to adherence to the preventive measures against COVID-19. A cut-off point was used to classify the practices into two levels: inadequate (<6) and adequate (≥6). This cut-off point was decided after identifying a mean adherence score value of 6, where those who scored lower values were indicated as having inadequate adherence and those who scored higher values were indicated as having adequate adherence.

Subsequently, inferential statistics were performed in two steps to test the variation in the level of adherence according to the sample characteristics. The first step included performing a paired t-test to assess the variations in the overall adherence levels as continuous variables before and after receiving the vaccines according to the measured sample’s sociodemographic characteristics. Additionally, the Chi-squared test was used to assess the variations in adherence to each practice item as a binary variable before and after receiving the vaccines. A value of *p* = 0.05 was considered statistically significant for the applied statistical tests.

Factors associated with adherence levels among the studied community were correlated with the health belief model to allow for the possible understanding of the barriers, norms, and drivers of adherence to personal protective behavior; disease-based factors that may affect adherence; population-based adherence, which may influence adherence; and finally, geography-based factors that might influence adherence.

## 3. Results

In total, 480 participants agreed to participate in the current investigation, with a total response rate of 97.6%. Table 1 illustrates the sociodemographic characteristics of the recruited sample. It may be noted that nearly 50% of the sample were above the age of 40, more than half were males (57.9%), and the majority were married and living with their spouses (78.1%). Furthermore, the majority of the sample indicated receiving a university education (74%) and reported living in urban areas of the Jazan region (67.9%). Nearly one-third of the sample indicated that they were healthcare workers (31.5%). The majority of the sample reported living with other persons in their residences (97.1%). Finally, less than half of the sample reported having a diagnosed chronic disease (43.5%), and a minority indicated that they were current smokers at the time of recruitment (13.8%).

Table 2 displays the distribution of the estimated levels of adherence to personal protective measures before and after receiving the COVID-19 vaccination, according to the measured study characteristics. The overall mean adherence score was 6.62 (SD: 2.9) before receiving the vaccine and 3.96 (SD: 2.2) after receiving the vaccine. The reduction in the mean of overall levels of adherence reached 2.66, and this reduction was statistically significant (*p* value < 0.001). This reduction in the overall mean adherence score suggests lower adherence among adults living in the Jazan region to the personal protective measures against COVID-19 after receiving the vaccination.

Before people received the COVID-19 vaccination, the mean level of adherence varied between 7.8 (the highest mean adherence level, found among smokers) and 5.77 (the lowest mean adherence level, found among the participants residing in rural areas). Although the current investigation did not investigate why smokers were more likely to report higher adherence scores, it can be postulated that the awareness of the smokers that they were at higher risk of developing serious respiratory complications of COVID-19 might encourage them to adhere to personal protective measures. Similarly, the lower adherence level score identified in rural areas can be partially explained by the lower probability of adults living in rural areas of the Jazan region being exposed to the enforcement of the precautionary measures applied by official institutions in the region (including nationwide precautionary measures, as indicated in Figure 1).

After people received the COVID-19 vaccination, the mean level of adherence varied between 5.78 (the highest mean adherence score, found among people who reported living alone) and 3.4 (the lowest mean adherence level, found among participants residing in rural areas). The reason for the higher adherence scores among those who lived alone, though not measured in the current investigation, can be postulated to be the fact that adults living alone might exhibit higher adherence to measures related to social isolation when experiencing symptoms suggesting COVID-19 infection, in comparison with those who are living with other family members.

A comparison of the adherence scores before and after receiving the COVID-19 vaccination, according to the measured sample’s sociodemographic characteristics, indicated a reduction in the adherence levels after receiving the vaccination in all comparison groups (*p* values < 0.001), except for the comparison made according to whether people lived alone or shared the residence with others, which did not exhibit a statistically significant change. When the reductions in the means of the adherence scores were compared according to the study population’s characteristics, a variation in the reduction in the means was noted.

The age group of 30–39 years displayed the largest reduction in the mean adherence level compared with other age groups. Females exhibited larger reductions in the mean adherence scores in comparison with males. Those who were not married showed lower reductions in the mean adherence levels in comparison with those who were married. Adults with university-level education showed larger reductions in comparison with those with lower than university-level education.

Adults working in the health sector exhibited greater reductions in the mean adherence scores in comparison with those not working in the health sector. Similarly, those living in rural areas showed greater reductions in comparison with those residing in urban areas. Smokers exhibited greater reductions in the means in comparison with non-smokers. Finally, adults diagnosed with a chronic disease showed greater reductions in comparison with those who reported not being diagnosed with a chronic disease.

Table 3 displays a comparison of the reported adherence to specific personal preventive measures before and after receiving the COVID-19 vaccine among the study’s participants. Overall, there was a statistically significant reduction (*p* < 0.05) in the level of adherence to behavioral personal preventive measures according to pre- and post-vaccination status in all 10 aspects. Before the vaccination, there was a higher level of adherence to most of the preventive behavioral measures in comparison with after receiving the vaccine.

The highest levels of adherence were reported to be associated with using a tissue, handkerchief, or elbow to cover coughs (83.5%); staying at home in the case of contact with a confirmed COVID-19 case (77.7%); staying at home in the case of experiencing influenza-like symptoms (75.2%); correctly using face masks (73.5%); and using face masks (72.7%). Lower adherence levels were reported to the remaining measures, including avoiding mass gatherings (66.7%), practicing social distancing (61.7%), performing regular hand washing/sanitizing (58.3%), and avoiding touching the mouth, eyes, and nose (47.1%). The lowest level of adherence was related to avoiding shaking hand (46.0%).

**Figure 2:**
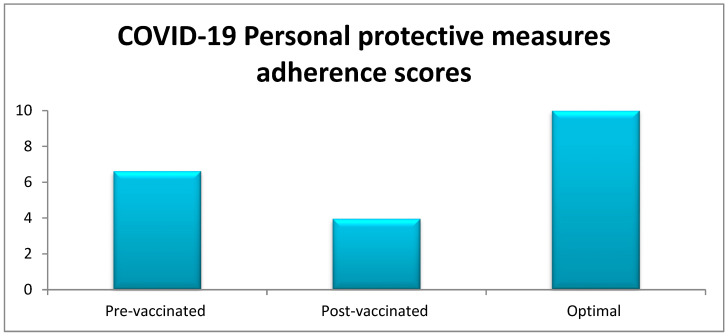
Optimal adherence scores regarding personal protective measures against COVID-19 and comparisons between the scores of pre-vaccinated and post-vaccinated participants in the Jazan region of Saudi Arabia.

After receiving the vaccination, there was a statistically significant decline in the level of adherence to all preventive behavioral measures (Figure 2). However, the highest levels of adherence were reported to be associated with using a tissue, handkerchief, or elbow to cover coughs (76.9%); staying at home in the case of contact with a confirmed COVID-19 case (65.8%); using a face mask (53.5%); correctly using a face mask (51.7%); and staying at home in the case of having influenza-like symptoms (42.9%). Lower adherence levels were indicated for practicing social distancing (40.4%); avoiding mass gatherings (28.3%); avoiding touching the mouth, eyes, and nose (25.0%); and avoiding shaking hand (8.8%). The lowest level of adherence was associated with regular hand washing/sanitizing (3.5%).

Although a high level of adherence remained for using a tissue, handkerchief, or elbow to cover coughs (76.9%), the level of adherence decreased sharply, by around 20% to 55%, for the remaining personal protective behaviors. The highest percentage of decrease in the level of adherence was related to regular hand washing/sanitizing (54.8%), followed by avoiding mass gatherings (38.4%), avoiding shaking hands (37.2%), and staying at home in the case of experiencing influenza-like symptoms (32.3%). Lower reductions were identified for avoiding touching the mouth, eyes, and nose (22.1%); correctly using a face mask (21.8%); practicing social distancing (21.3%); using a face mask (19.2%); and staying at home in the case of contact with a confirmed COVID-19 case (11.9%). The lowest percentage of decrease in the level of adherence (6.6%) was reported to be associated with using a tissue, handkerchief, or elbow to cover coughs.

Table 4 illustrates the correlations among the factors associated with adherence to personal protective measures against COVID-19 according to vaccination status and the health belief model. Vaccination status was associated with lower adherence to some preventive behavioral measures, including hand hygiene and avoiding close contact with sick people, possibly due to a false sense of security among vaccinated individuals. The factors identified by the current investigation as being associated with a reduction in adherence were classified into two domains. The first domain was related to predictors of behavioral change according to the health belief model, namely, perceived susceptibility, perceived severity, perceived benefits, and perceived barriers. Furthermore, the second domain was related to the nature of the determinants of adherence, classified according to the disease, the population, and the geography.

After applying the health belief model to the constructs of the current investigation, we presumed that disease-based factors and population-based factors were associated with the perceived susceptibility, the perceived severity, and perceived health benefits. However, geography-based factors can be postulated to be associated with the perceived susceptibility only. The majority of the factors displayed in Table 4 can assumed to be motivators for adherence to personal protective measures against COVID-19, both before and after receiving the vaccine. However, being diagnosed with a chronic disease appeared to produce lower motivation for adherence, while education seemed to offer produce motivation or had no effect on the adherence level. Only the factor of being in a relationship seemed to have a motivating effect on adherence during the pre-vaccination stage but not after the vaccination, indicating the modifying effect of receiving the vaccine on the motivation for adherence.

## 4. Discussion

This study involved a cross-sectional investigation conducted in Jazan, Saudi Arabia, to assess adherence levels to preventive behavioral measures against COVID-19 among the community, according to their COVID-19 vaccination status. The findings indicated that the adherence to preventive measures by the participants was higher before receiving the vaccination than after. This suggests a change in the behavior of the community members toward COVID-19 due to receiving the vaccination and a reduction in adherence to respiratory hygiene practices.

The findings of our current investigation can be compared with those of similar assessments. Some previous studies have shown that vaccinated people may reduce their precautionary behavior regarding COVID-19 [11]. This would represent a real threat to controlling this epidemic and justifies investment into health education endeavors. Feeling safe and avoiding danger may induce a desire to let go of precautionary measures [12]. A comparison of this study’s findings to those of similar studies assessing adherence to COVID-19 preventive measures according to vaccination status revealed similarities. In a study in India by Nizam et al., it was concluded that adherence to COVID-19 preventive measures was reduced after vaccination [13]. On the other hand, a study by Wright et al. conducted in the UK revealed contradictory findings, where an increase in the adherence level was noted even after receiving the COVID-19 vaccination [14]. Nonetheless, it can be postulated that the epidemiology of COVID-19 in the UK and the occurrence of a second wave of the disease might partially explain the extended period of adherence, even after receiving the vaccine.

All preventive behavioral practices were reduced post-vaccination, where the largest reduction was related to the practice of hand washing and the use of sanitizers, followed by the avoidance of mass gatherings and shaking hands. These results are compatible with those of previous studies in Egypt, Nigeria, and Ethiopia [15,16]. This indicates that not all COVID-19 preventive measures are given equal attention. Long-term commitment is vital to mitigate the spread of the disease and reduce its impact.

The assessment of factors associated with adherence to COVID-19 preventive behavioral measures in the current investigation after vaccination suggested that being 50 years old or older, female, a healthcare worker, and a smoker were associated with higher adherence levels compared with other groups in the same categories. Elderly people are more vulnerable to complications from COVID-19 and are thus more committed to preventive precautions, as expected [17].

A survey by Almalki recruited a sample of 597 respondents from Jazan and assessed their knowledge, attitudes, and practices concerning COVID-19 during May 2020, indicating an adequate level of knowledge, a good attitude, and acceptable practices regarding the disease [18]. However, an assessment of practices revealed that females had greater odds of reporting wearing a face mask compared with males. Considering the differences in the scope of Almalki’s study and the current study, Almalki did not assess the influence of vaccination status, unlike in our research, and females exhibited higher adherence levels in the current investigation both before and after vaccination against COVID-19.

Previous studies have shown that low levels of education, occupation, and income are associated with reduced adherence to preventive measures [19,20]. The high level of knowledge among healthcare workers may be a crucial factor contributing to the high levels of adherence to preventive measures [21]. Smokers are probably more adherent to preventive measures, since their lungs are already compromised by smoking; therefore, they have a lower chance of surviving COVID-19 than healthy people [22].

According to a review by Sabaté, lack of access to healthcare, financial constraints, side effects of medication, forgetfulness, and competing priorities can act as barriers to adherence to medication. Other factors such as cultural, social, and family beliefs, attitudes, and values can influence adherence norms. In addition, the perceived benefits of treatment, social support, and healthcare–provider communication and trust are some of the drivers of adherence [23]. Disease-based factors, including the severity and chronicity of the disease, the complexity of the treatment regimen, co-morbidities, and the presence of symptoms, can all affect adherence [24]. Population-based factors, such as age, gender, race, and socioeconomic status, as well as access to healthcare and education, can also impact adherence. Health literacy and self-efficacy are other factors that may play a role in adherence [25]. Geography-based factors, including geographic location (such as rural or urban areas), and access to healthcare and transportation, as well as environmental factors such as air pollution and climate can also influence adherence [26]. Improved health outcomes and disease management, reduced healthcare costs and burden on the healthcare system, improved quality of life and productivity, increased patient satisfaction, and trust in healthcare providers are some of the positive consequences of adherence [23].

It can be noted that adherence usually refers to the uptake of a particular medication or vaccine. However, our investigation studies the effect of receiving an intervention, namely the COVID-19 vaccine, on adherence levels. As indicated in Table 4, the motivators of adherence are explained by the perceived susceptibility, perceived severity, and perceived benefit, depending on the nature of the disease, the type of the population, and the geography, whereby several explanations can be provided for the detected associations.

For example, people with relationships were noted to exhibit higher adherence levels before receiving the vaccine but not afterward. This can be associated with the reduced perceived benefit of adherence to personal protective behavior for protecting family members after receiving the vaccine. Additionally, it can be postulated that healthcare workers were more motivated to adhere to personal protective measures, both before and after receiving the vaccine, due to their perceived susceptibility and because they were at a higher risk of infection, in addition to adherence to protective measures being a requirement of their work conditions.

Perceived disease severity can partially explain the higher adherence levels among smokers and older participants, as they are at a higher risk of developing the severe consequences of the infection due to the nature of the disease and due to their personal characteristics. The higher adherence levels among those in relationships may suggest the presence of perceived benefits, as adherence to protective measures is likely to protect family members against the infection. Nonetheless, the perceived benefit of adherence was no longer present after receiving the vaccination, suggesting the presence of a behavior modification that can be partially correlated with receiving the vaccine. Nonetheless, the lower motivation among people with chronic diseases to adhere to personal preventive measures requires further assessment. Geographical determinants can be postulated to be present regarding the motivation of people in urban areas to exhibit higher adherence levels before and after receiving the vaccine due to their higher susceptibility to infection in comparison with those living in less crowded urban areas.

The current study has multiple areas of strengths and weaknesses. The main strength of the current investigation is related to the timing of the data collection and its ability to reach a sample of vaccinated individuals to enable an assessment of the adherence to preventive measures against COVID-19 before the lifting of preventive precautionary measures against COVID-19 in Saudi Arabia. Another strength area is related to the behavioral conceptualization applied in the current study and the potential ability to generalize the findings of the study to other international contexts or future pandemics of similar respiratory illnesses. The main limitation of the current investigation is related to the design, which was reliant on retrospective data collection via a cross-sectional design. Nonetheless, it is possible to argue that the practices assessed before and after receiving the vaccination were measured within a relatively short timeframe and the probability of a recall bias, though it cannot be neglected, is low.

## 5. Conclusions

The assessment of the levels of adherence to COVID-19 preventive measures among the community according to COVID-19 vaccination status revealed that adherence to preventive behavioral measures was higher among the participants before receiving the vaccination than afterward. All preventive behavioral practices were reduced after vaccination; the largest reduction was related to hand washing practices and the use of sanitizers, followed by the avoidance of mass gatherings and shaking hand. This indicates the importance of raising awareness about the possibility of re-infection with COVID-19 despite having been vaccinated, and increasing awareness about preventive behavioral measures, especially with the ongoing incidence of COVID-19 cases. Furthermore, this investigation indicates the need for the development of public health policies that incorporate prevention measures being maintained after exposure to a particular intervention (such as vaccination), with measures aiming to enhance overall personal hygiene (such as respiratory hygiene) in order to increase the effectiveness of the applied public health interventions in real-life situations.

## Figures and Tables

**Table 1:** Sociodemographic characteristics of a sample of 480 participants from Jazan, Saudi Arabia.

Item	Frequency (Proportion)
Age (years)	
18–29	93 (19.4%)
30–39	146 (30.4%)
40–49	118 (24.6%)
≥50	123 (25.6%)
Gender	
Male	278 (57.9%)
Female	202 (42.1%)
Marital status	
Single/divorced/widowed	105 (21.9%)
Married/living together	375 (78.1%)
Education	
≤Secondary	122 (25.4%)
≥University	358 (74.6%)
Belonging to the health sector	
No	329 (68.5%)
Yes	151 (31.5%)
Area of residency	
Urban	326 (67.9%)
Rural	154 (32.1%)
Living conditions	
Living with others	466 (97.1%)
Living alone	14 (2.9%)
Smoking	
Smoker	66 (13.8%)
Non-smoker	414 (86.3%)
Having a chronic disease	
With a chronic disease	209 (43.5%)
No chronic disease	271 (56.5%)

**Table 2:** Comparison of pre- and post-COVID-19 vaccination levels of adherence to personal protective measures among 480 participants in the Jazan region according to their sociodemographic characteristics.

Item	N (%)	Adherence Level			
		Pre-Vaccination	Post-Vaccination	Mean Reduction	*p* Value *
		Mean ± SD	Mean ± SD		
Age (years)					
18–29	93 (19.4)	6.47 ± 2.89	3.75 ± 2.37	−2.72	*p* < 0.001
30–39	146 (30.4)	6.68 ± 2.93	3.78 ± 2.11	−2.9	*p* < 0.001
40–49	118 (24.6)	6.59 ± 3.17	3.88 ± 2.43	−2.71	*p* < 0.001
≥50	123 (25.6)	6.70 ± 2.65	4.44 ± 2.08	−2.26	*p* < 0.001
Gender					
Male	278 (57.9)	6.21 ± 2.95	3.73 ± 2.26	−2.48	*p* < 0.001
Female	202 (42.1)	7.20 ± 2.76	4.29 ± 2.19	−2.91	*p* < 0.001
Marital status					
Single/divorced/widowed	105 (21.9)	6.52 ± 2.80	4.10 ± 2.58	−2.42	*p* < 0.001
Married/living together	375 (78.1)	6.65 ± 2.94	3.93 ± 2.15	−2.72	*p* < 0.001
Education					
≤Secondary	122 (25.4)	6.17 ± 2.98	3.98 ± 2.04	−2.19	*p* < 0.001
≥University	358 (74.6)	6.77 ± 2.87	3.96 ± 2.31	−2.81	*p* < 0.001
Belonging to the health sector					
No	329 (68.5)	6.38 ± 2.96	3.75 ± 2.22	−2.63	*p* < 0.001
Yes	151 (31.5)	7.15 ± 2.71	4.44 ± 2.23	−2.71	*p* < 0.001
Area of residency					
Urban	326 (67.9)	7.02 ± 2.69	4.24 ± 2.23	−2.78	*p* < 0.001
Rural	154 (32.1)	5.77 ± 3.17	3.40 ± 2.17	−2.37	*p* < 0.001
Live alone or with other people					
Live alone	14 (2.9)	6.86 ± 3.41	5.78 ± 2.51	−1.08	*p* = 0.119
Smoking					
Smoker	66 (13.8)	7.80 ± 2.18	4.44 ± 2.44	−3.36	*p* < 0.001
Non-smoker	414 (86.3)	6.44 ± 2.96	3.89 ± 2.2	−2.55	*p* < 0.001
Having a chronic disease					
With a chronic disease	209 (43.5)	6.33 ± 3.14	3.60 ± 2.34	−2.73	*p* < 0.001
No chronic disease	271 (56.5)	6.84 ± 2.7	4.25 ± 2.13	−2.59	*p* < 0.001
Overall adherence score (OALS)		6.62 ± 2.9	3.96 ± 2.2	−2.66	2.66

* Paired t test.

**Table 3:** Comparison of pre- and post-COVID-19 vaccination levels of adherence to personal protective measures among 480 participants in the Jazan region.

Item	Pre-Vaccination	Post-Vaccination	% Change	*p* Value *
	N (%)	N (%)		
Physical distancing 1.5–2 m				
Yes	296 (61.7)	194 (40.4)	−21.3	*p* < 0.001
No	184 (38.3)	286 (59.6)		
Use of face mask				
Yes	349 (72.7)	257 (53.5)	−19.2	*p* < 0.001
No	131 (27.3)	223 (46.5)		
Correct mask use				
Yes	353 (73.5)	248 (51.7)	−21.8	*p* < 0.001
No	127 (26.5)	232 (48.3)		
Regular hand washing and alcohol-based hand sanitizer use				
Yes	280 (58.3)	17 (3.5)	−54.8	*p* = 0.002
No	200 (41.7)	463 (96.5)		
Avoiding handshakes				
Yes	221 (46)	42 (8.8)	−37.2	*p* < 0.001
No	259 (54)	438 (91.3)		
Avoiding touching the mouth, eyes, and nose				
Yes	226 (47.1)	120 (25)	−22.1	*p* < 0.001
No	254 (52.9)	360 (75)		
Coughing by covering mouth/nose with a disposable handkerchief or coughing into the crease of the elbow				
Yes	401 (83.5)	369 (76.9)	−6.6	*p* < 0.001
No	79 (16.5)	111 (23.1)		
Staying at home in the case of influenza-like symptoms				
Yes	361 (75.2)	206 (42.9)	−32.3	*p* < 0.001
No	119 (24.8)	274 (57.1)		
Stay at home in the case of contact with a COVID-19 case				
Yes	373 (77.7)	316 (65.8)	−11.9	*p* < 0.001
No	107 (22.3)	164 (34.2)		
Avoiding mass gathering				
Yes	320 (66.7)	136 (28.3)	−38.4	*p* < 0.001
No	160 (33.3)	344 (71.7)		

* Chi-squared test.

**Table 4:** Correlations among factors associated with adherence to personal protective measures against COVID-19 according to vaccination status and the health belief model.

	Nature of the Determinants of Adherence			Factors Assessed According to Vaccination Status	
Behaviour Predictors	Disease	Population	Geography	Pre-Vaccination	Post-Vaccination
Perceived susceptibility	√	√	√	↑ Females ↑ People in urban areas ↑ Healthcare workers	↑ Females ↑ People in urban areas ↑ Healthcare workers
Perceived severity	√	√		↑ Smokers ↑ Older age groups ↓ People with chronic diseases	↑ Smokers ↑ Older age groups ↓ People with chronic diseases
Perceived benefits	√	√		↓ People with higher education ↑ People in relationships	↔ People with higher education ↓ People in relationships

↑: suggests higher motivation. ↓: suggests lower motivation. ↔: suggests no effect on motivation.

## Data Availability

Data are available upon request due to the ethical restrictions regarding the participants’ privacy. Requests for the data may be sent to the corresponding author.
